# Supplementary material for: Distinct genetic signals at the FGF21 locus complicate studies of FGF21's role in diet regulation using human cohort data
Source: Mol Metab. 2024 Oct 18;90:102049. doi: 10.1016/j.molmet.2024.102049 (PMC11546929; doi:10.1016/j.molmet.2024.102049)
Supplement: Multimedia component 1 [file mmc1.pdf]

Distinct genetic signals at the *FGF21* locus complicate studies of FGF21's role in diet regulation using human cohort data

Stina Ramne[1], Mario García-Ureña[1], Matthew P Gillum[1,2], Lars Ängquist[1], Torben Hansen[1], Jordi Merino[1,3,4,5], Niels Grarup[1]*

[1]Novo Nordisk Foundation Center for Basic Metabolic Research, Faculty of Health and Medical Sciences, University of Copenhagen, Copenhagen, Denmark

[2]Novo Nordisk A/S, Early Innovation, Outreach, and Alliances, Måløv, Denmark

[3]Novo Nordisk Foundation Center for Genomic Mechanisms of Disease, Broad Institute of MIT and Harvard, Cambridge, MA, USA

[4]Diabetes Unit, Endocrine Division, Massachusetts General Hospital, Boston, MA, USA

[5]Center for Genomic Medicine, Massachusetts General Hospital, Boston, MA, USA

*Correspondence: Niels Grarup, niels.grarup@sund.ku.dk

Supplementary table 1. *CARMA* fine-mapping of the region of chr19:49180000 to 49300000 for FGF21, protein intake, carbohydrate intake and fat intake.

Trait	Chr	Position	A1	A0	EAF	rsID	Consequence	Beta	SE	P-value	PIP
FGF21	19	49249663	G	C	0.04	rs28400017	Intron variant	-0.152	0.019	2.4E-15	0.711
FGF21	19	49260677	A	C	0.54	rs838131	Intron variant	0.123	0.008	4.7E-58	1.000
FGF21	19	49261038	G	A	0.30	rs1688263	Intron variant	-0.108	0.008	7.1E-39	0.906
FGF21	19	49264232	C	T	0.09	rs79390987	Downstream gene variant	-0.085	0.013	1.8E-10	0.997
FGF21	19	49273784	G	A	0.30	rs560343	Intergenic variant	-0.084	0.008	3.6E-25	1.000
Protein	19	49259529	A	G	0.44	rs838133	Synonymous variant	-0.109	0.010	2.3E-27	1.000
Protein	19	49261368	T	C	0.39	rs739320	Missense variant	-0.103	0.011	2.8E-22	0.166
Fat	19	49218060	T	C	0.48	rs35866622	Splice region variant	-0.176	0.019	6.5E-21	0.305
Fat	19	49218111	T	G	0.53	rs33988101	Synonymous variant	-0.183	0.019	1.4E-22	0.633
Fat	19	49259529	A	G	0.44	rs838133	Synonymous variant	-0.198	0.020	1.6E-23	1.000
Carbo-hydrate	19	49259529	A	G	0.44	rs838133	Synonymous variant	0.210	0.025	2.4E-17	0.994

Results of *CARMA* fine-mapping, listing genetic variants that are genome-wide significant ($P<5\times10^{8}$) and with a PIP >0.1.

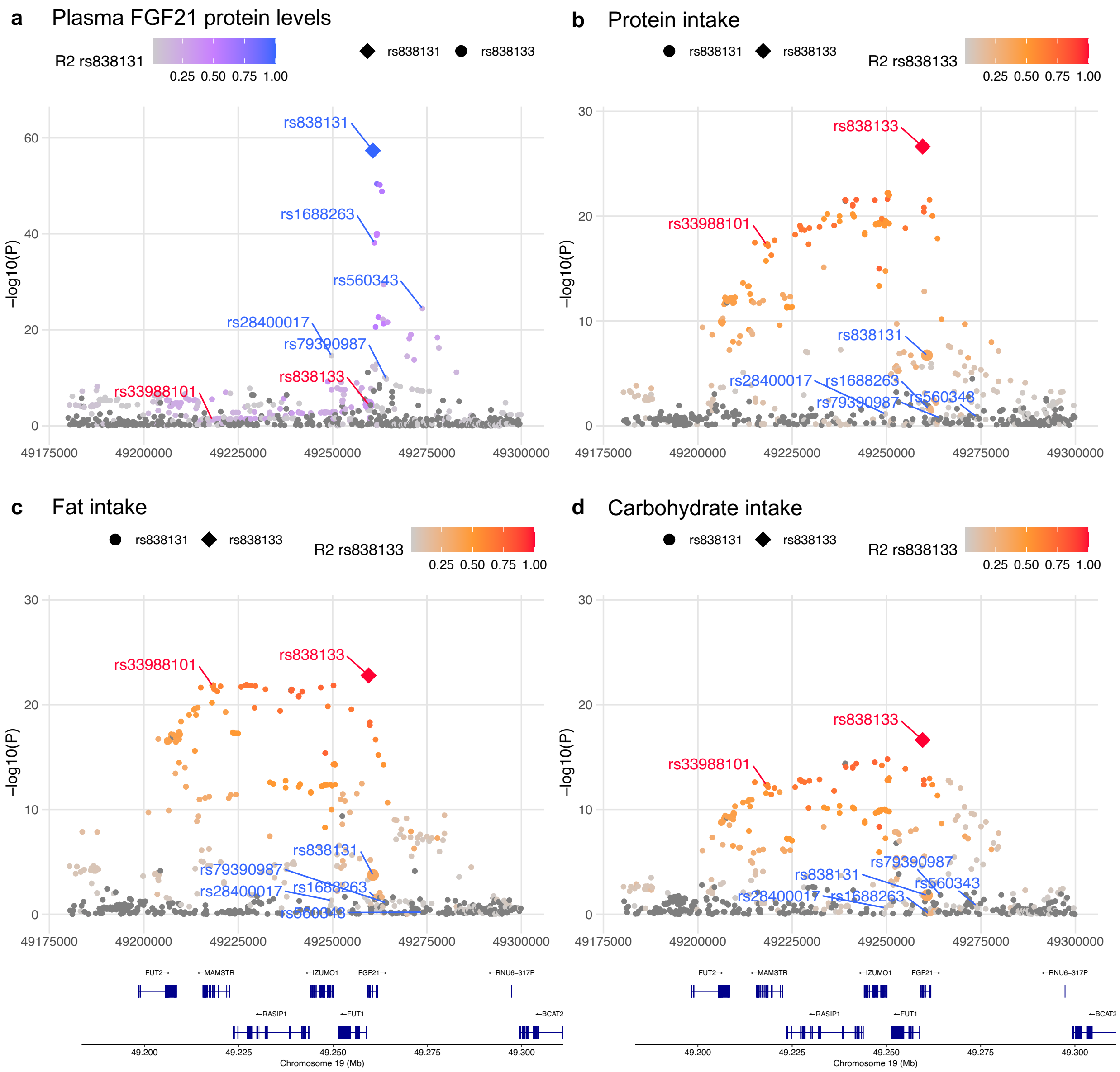

Supplementary figure 1. Regional plots of the *FGF21* region 19:49,180,000 to 19:49,300,000 for **a**, plasma FGF21 protein levels, **b**, protein intake, **c**, fat intake and **d**, carbohydrate intake, highlighting probable causal variants (PIP>0.5) identified with *CARMA*. Variants in blue are identified as probable causal variants for plasma FGF21 protein levels and variants in red are identified as probable causal variants for macronutrient intake (rs838133 is identified for protein, fat and carbohydrate, while rs33988101 is only identified for fat).

Supplementary table 2. Results of colocalization analyses between plasma FGF21 levels and macronutrient intake in the region of chr19:49180000 to 49300000.

		**Merino 2022**			**Merino 2018**		
		**CHARGE+UKBB, n=282,271**			**CHARGE, n=91,114**		
		Protein intake	Carbo-hydrate intake	Fat intake	Protein intake	Carbo-hydrate intake	Fat intake
	N snps	609	609	609	368	368	368
**H0.PP**	No association with either trait	2.9E-71	3.3E-61	1.2E-67	5.4E-54	1.7E-53	1.9E-53
**H1.PP**	Association with trait 1, not with trait 2	1.1E-20	1.3E-10	4.5E-17	2.1E-03	6.6E-03	7.6E-03
**H2.PP**	Association with trait 2, not with trait 1	2.6E-51	2.6E-51	2.6E-51	2.6E-51	2.6E-51	2.6E-51
**H3.PP**	Association with trait 1 and trait 2, two independent SNPs	1.00	1.00	1.00	0.99	0.99	0.99
**H4.PP**	Association with trait 1 and trait 2, one shared SNP	1.8E-17	4.8E-12	9.7E-17	6.4E-03	2.6E-03	2.6E-03

Colocalization was analyzed using the *coloc* R-package using the default prior probabilities.

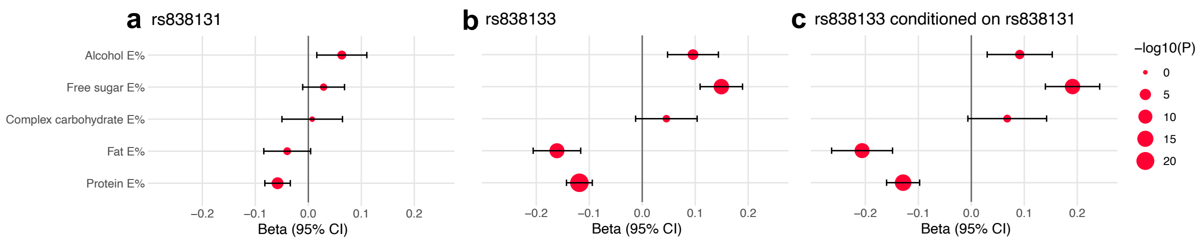

Supplementary figure 2. Association between *FGF21* genetic variants and macronutrient intake. Analyses are adjusted for age, sex, study center, BMI, total energy intake and 10 genetic PCs (n=146,241-157,508).

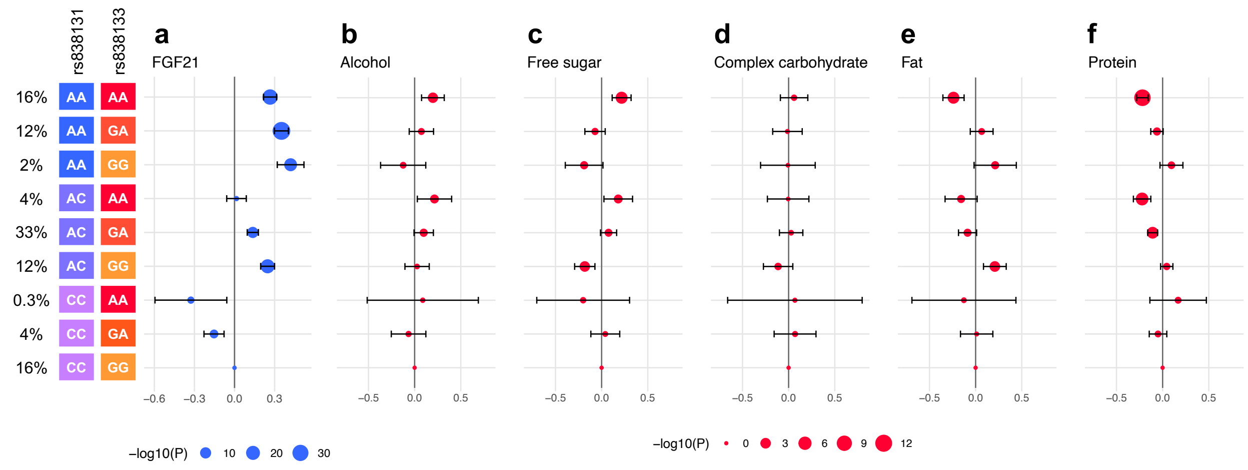

Supplementary figure 3. Association between rs838131 and rs838133 genotype group combinations and plasma FGF21 levels and macronutrient intake levels. **a**, Analyses with plasma FGF21 are adjusted for age, sex, study center, fasting time, BMI and 10 genetic PCs (n=36,029). **b-f**, Analyses with macronutrients are adjusted for age, sex, study center, BMI, total energy intake and 10 genetic PCs (n=146,241).

Supplementary table 3. Association between macronutrient substitutions and plasma FGF21 protein levels stratified by BMI≥30 or BMI<30.

**BMI ≥30, n=4585**	**Beta**	**SE**	**P**	**95% CI L**	**95%CI U**
Alcohol vs protein	0.047	0.006	5.3E-16	0.059	0.036
Alcohol vs fat	0.016	0.004	1.5E-05	0.023	0.009
Alcohol vs complex carbohydrate	0.022	0.003	1.4E-13	0.028	0.016
Alcohol vs free sugar	0.018	0.004	1.9E-05	0.027	0.010
Free sugar vs protein	0.028	0.005	2.4E-07	0.039	0.017
Free sugar vs fat	-0.003	0.004	4.4E-01	0.005	-0.012
Free sugar vs complex carbohydrate	0.004	0.004	2.4E-01	0.011	-0.003
Complex carbohydrate vs protein	0.024	0.006	2.9E-05	0.035	0.013
Complex carbohydrate vs fat	-0.008	0.003	1.5E-02	-0.002	-0.014
Fat vs protein	0.032	0.006	2.5E-07	0.044	0.020
**BMI <30, n=17019**					
Alcohol vs protein	0.064	0.003	7.4E-88	0.070	0.058
Alcohol vs fat	0.031	0.002	2.5E-58	0.034	0.027
Alcohol vs complex carbohydrate	0.036	0.002	1.1E-121	0.039	0.033
Alcohol vs free sugar	0.022	0.002	1.2E-23	0.026	0.017
Free sugar vs protein	0.041	0.003	3.2E-40	0.047	0.035
Free sugar vs fat	0.007	0.002	1.5E-03	0.012	0.003
Free sugar vs complex carbohydrate	0.014	0.002	3.3E-13	0.018	0.010
Complex carbohydrate vs protein	0.026	0.003	3.6E-16	0.032	0.020
Complex carbohydrate vs fat	-0.008	0.002	8.9E-06	-0.004	-0.011
Fat vs protein	0.033	0.003	7.8E-23	0.040	0.027

Analyses were adjusted for age, sex, study center, fasting time and total energy intake in addition to macronutrients for substitution modelling.

Supplementary table 4. Association between macronutrient substitutions and plasma FGF21 protein levels stratified by fasting time ≥6 h or <6 h.

**Fasting time ≥6 h, n=2108**	**Beta**	**SE**	**P**	**95% CI L**	**95%CI U**
Alcohol vs protein	0.048	0.008	7.2E-09	0.064	0.032
Alcohol vs fat	0.030	0.005	5.6E-10	0.040	0.021
Alcohol vs complex carbohydrate	0.033	0.004	5.7E-17	0.041	0.026
Alcohol vs free sugar	0.024	0.006	2.7E-05	0.034	0.013
Free sugar vs protein	0.023	0.008	3.8E-03	0.038	0.007
Free sugar vs fat	0.005	0.006	3.9E-01	0.017	-0.006
Free sugar vs complex carbohydrate	0.010	0.005	4.7E-02	0.019	0.000
Complex carbohydrate vs protein	0.012	0.008	1.3E-01	0.029	-0.004
Complex carbohydrate vs fat	-0.005	0.005	2.4E-01	0.004	-0.014
Fat vs protein	0.018	0.009	4.3E-02	0.035	0.001
**Fasting time <6 h, n=17019**					
Alcohol vs protein	0.067	0.003	3.3E-116	0.073	0.061
Alcohol vs fat	0.027	0.002	1.8E-52	0.030	0.023
Alcohol vs complex carbohydrate	0.032	0.001	1.0E-114	0.035	0.029
Alcohol vs free sugar	0.021	0.002	1.5E-25	0.025	0.017
Free sugar vs protein	0.045	0.003	1.7E-58	0.050	0.039
Free sugar vs fat	0.004	0.002	4.3E-02	0.008	0.000
Free sugar vs complex carbohydrate	0.011	0.002	2.4E-10	0.015	0.008
Complex carbohydrate vs protein	0.033	0.003	2.4E-30	0.039	0.027
Complex carbohydrate vs fat	-0.008	0.002	1.2E-06	-0.005	-0.011
Fat vs protein	0.041	0.003	2.4E-39	0.047	0.035

Analyses were adjusted for age, sex, study center, BMI and total energy intake in addition to macronutrients for substitution modelling.

Supplementary table 5. Association between macronutrient substitutions and plasma FGF21 protein levels adjusting for *FGF21* genetic variation.

**Unadjusted**	**Beta**	**SE**	**P**	**95% CI L**	**95%CI U**
Alcohol vs protein	0.066	0.003	3.9E-87	0.060	0.073
Alcohol vs fat	0.027	0.002	3.0E-43	0.023	0.030
Alcohol vs complex carbohydrate	0.035	0.002	5.2E-99	0.032	0.039
Alcohol vs free sugar	0.023	0.002	6.9E-22	0.019	0.028
Free sugar vs protein	0.040	0.003	2.4E-41	0.034	0.046
Free sugar vs fat	0.003	0.002	0.176	-0.001	0.008
Free sugar vs complex carbohydrate	0.011	0.002	1.8E-08	0.007	0.015
Complex carbohydrate vs protein	0.029	0.003	3.9E-20	0.023	0.035
Complex carbohydrate vs fat	-0.008	0.002	7.8E-07	-0.012	-0.005
Fat vs protein	0.039	0.004	6.3E-29	0.033	0.046
**Adjusted for rs838131**					
Alcohol vs protein	0.065	0.003	6.0E-86	0.059	0.072
Alcohol vs fat	0.026	0.002	3.6E-41	0.022	0.030
Alcohol vs complex carbohydrate	0.035	0.002	1.9E-97	0.032	0.038
Alcohol vs free sugar	0.023	0.002	7.9E-22	0.019	0.028
Free sugar vs protein	0.039	0.003	1.5E-40	0.034	0.045
Free sugar vs fat	0.002	0.002	0.271	-0.002	0.007
Free sugar vs complex carbohydrate	0.011	0.002	3.6E-08	0.007	0.015
Complex carbohydrate vs protein	0.028	0.003	6.4E-20	0.022	0.034
Complex carbohydrate vs fat	-0.009	0.002	2.7E-07	-0.012	-0.005
Fat vs protein	0.039	0.004	3.3E-29	0.033	0.046
**Adjusted for rs838133**					
Alcohol vs protein	0.066	0.003	1.1E-85	0.059	0.072
Alcohol vs fat	0.026	0.002	1.6E-41	0.022	0.030
Alcohol vs complex carbohydrate	0.035	0.002	2.9E-97	0.032	0.038
Alcohol vs free sugar	0.023	0.002	2.3E-21	0.018	0.028
Free sugar vs protein	0.040	0.003	8.3E-41	0.034	0.046
Free sugar vs fat	0.003	0.002	0.209	-0.002	0.007
Free sugar vs complex carbohydrate	0.011	0.002	2.0E-08	0.007	0.015
Complex carbohydrate vs protein	0.028	0.003	7.8E-20	0.022	0.035
Complex carbohydrate vs fat	-0.008	0.002	4.2E-07	-0.012	-0.005
Fat vs protein	0.039	0.004	6.5E-29	0.033	0.046
**Adjusted for rs838131 and rs838133**					
Alcohol vs protein	0.066	0.003	3.4E-88	0.060	0.073
Alcohol vs fat	0.026	0.002	8.9E-42	0.022	0.030
Alcohol vs complex carbohydrate	0.035	0.002	3.4E-97	0.032	0.038
Alcohol vs free sugar	0.023	0.002	2.3E-21	0.018	0.028
Free sugar vs protein	0.040	0.003	1.2E-42	0.035	0.046
Free sugar vs fat	0.003	0.002	0.196	-0.002	0.007
Free sugar vs complex carbohydrate	0.011	0.002	2.0E-08	0.007	0.015

Complex carbohydrate vs protein	0.029	0.003	4.5E-21	0.023	0.035
Complex carbohydrate vs fat	-0.008	0.002	5.6E-07	-0.012	-0.005
Fat vs protein	0.040	0.004	3.4E-30	0.033	0.047
**Adjusted for weigthed SNP-score of 7 pQTLs**					
Alcohol vs protein	0.066	0.003	3.4E-85	0.060	0.073
Alcohol vs fat	0.026	0.002	4.6E-40	0.022	0.030
Alcohol vs complex carbohydrate	0.035	0.002	1.2E-93	0.032	0.038
Alcohol vs free sugar	0.024	0.002	3.5E-22	0.019	0.029
Free sugar vs protein	0.040	0.003	7.4E-40	0.034	0.046
Free sugar vs fat	0.002	0.002	0.376	-0.002	0.007
Free sugar vs complex carbohydrate	0.010	0.002	2.6E-07	0.006	0.014
Complex carbohydrate vs protein	0.029	0.003	2.1E-20	0.023	0.035
Complex carbohydrate vs fat	-0.008	0.002	6.5E-07	-0.012	-0.005
Fat vs protein	0.040	0.004	2.5E-29	0.033	0.047

Analyses were adjusted for age, sex, study center, fasting time, BMI, total energy intake and 10 genetics PCs in addition to macronutrients for substitution modelling and the indicated genetic variants.

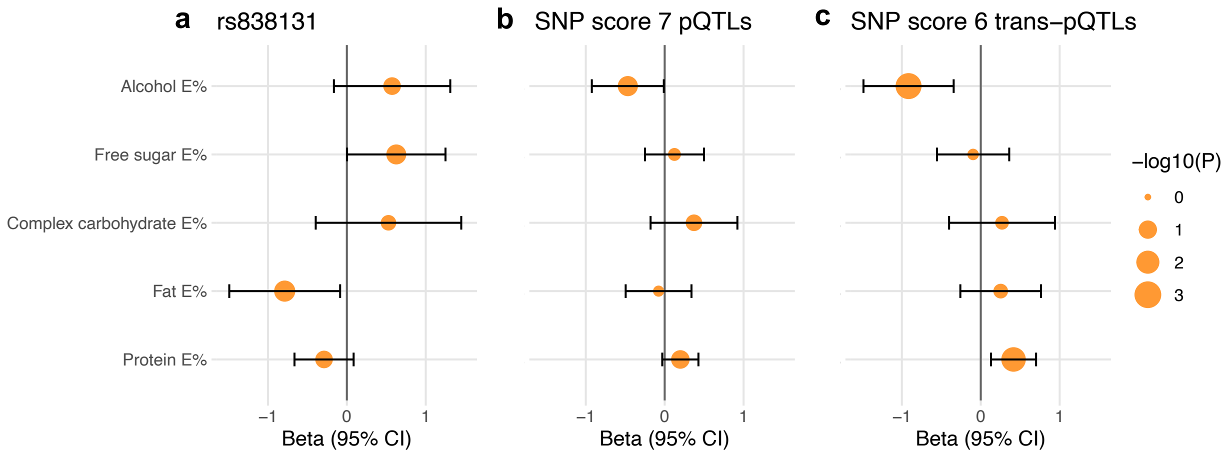

Supplementary figure 4. One-sample MR analyzes estimating potentially causal effects of plasma FGF21 on macronutrient intake using different IVs: **a**, the FGF21 cis-pQTL (rs838131), **b**, all 7 FGF21 pQTLS composed into a weighted score, and **c**, only the 6 trans-pQTLs (excluding rs838131) composed into a weighted score. Analyses were adjusted for age, sex, study center, fasting time, BMI, total energy intake and 10 genetic PCs (n=15,512-16,400).

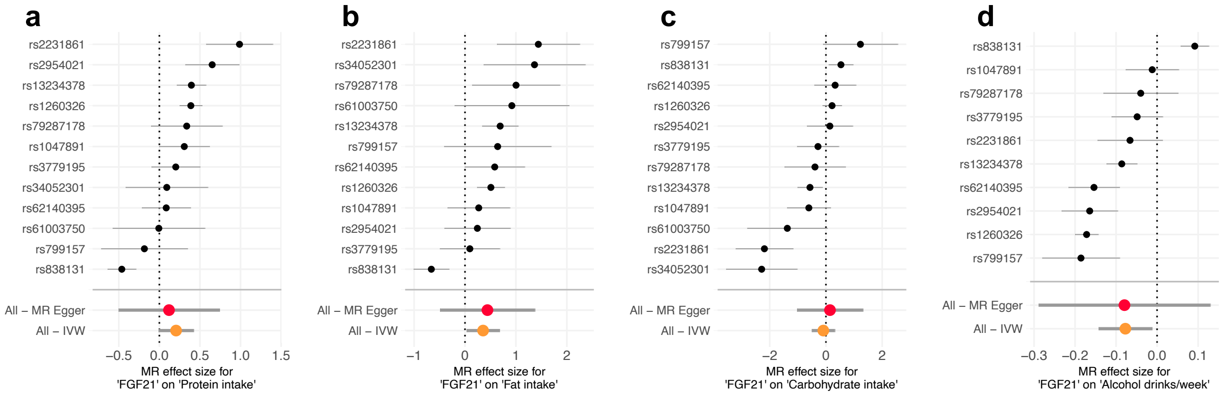

Supplementary figure 5. Forest plot of single IV estimates in two-sample MR estimating the potentially causal effect of plasma FGF21 on **a**, protein intake, **b**, fat intake, **c**, carbohydrate intake, and **d**, alcohol intake.

Supplementary table 6. Two-sample MR heterogeneity when including or excluding the rs838131 variant as IV.

	All IVs				Excluding rs838131			
	Q	df	P	i^2	Q	df	P	i^2
Protein								
IVW	93.1	11	4.2E-15	88.2%	23.4	10	9.2E-03	57.4%
Egger	92.3	10	1.9E-15	89.2%	23.4	9	5.5E-03	61.5%
Fat								
IVW	53.6	11	1.4E-07	79.5%	13.3	10	2.1E-01	24.7%
Egger	53.4	10	6.3E-08	81.3%	11.4	9	2.5E-01	21.2%
Carbohydrate								
IVW	54.3	11	6.3E-08	79.8%	44.2	10	2.7E-06	77.4%
Egger	53.4	10	1.0E-07	81.3%	44.5	9	1.3E-06	79.8%
Alcohol								
IVW	160.1	8	7.1E-30	95.0%	41.3	8	2.4E-06	80.6%
Egger	160.1	9	1.5E-30	94.4%	38.6	7	1.8E-06	81.8%

Heterogeneity was tested with the *mr_heterogeneity* function for both the IVW model and the MR Egger model. I^2 was calculated as 100*(Q-df)/Q.
